# Hysteria

**Published:** 1907-08-31

**Authors:** 


### Illustrative Cases.

### HYSTERIA.

The following case is one which well illustrates the danger of diagnosing hysteria as the cause of symptoms in cases where an organic lesion cannot be excluded with absolute certainty.

Hysteria is a very easy diagnosis to make, but it is by no means easy to make it correctly. It is really only when every possible organic cause for the symptoms has been absolutely put out of court that hysteria should be diagnosed; it is a very unfortunate occurrence for the patient to die shortly after a diagnosis of hysteria has been made. It is too easy to jump to the conclusion that symptoms are purely functional, but in reality the jump is fraught with danger if it is made without due consideration; even when great care has been exercised there is sometimes a serious organic lesion when none whatever could be found to account for the symptoms. One of the very hardest diagnoses to make, probably, is that of "a perfectly healthy man," for it implies that every conceivable disease has been excluded; it is true that a very brief survey of the general appearances of a patient, an investigation of the physical signs, and an examination of the urine, enable one to form a very shrewd guess that the patient is perfectly sound, and the guess will again and again be right; this is so in examinations for life insurance, for example; but now and then people who have seemed quite healthy die within a year or two of diseases, such as tuberculosis, cirrhosis, or cerebral atheroma, which were actually in existence when the medical examination was made. Similarly, in the case of hysteria, one may turn out right time after time in calling the symptoms functional; but it is a thing to be chary of doing without good grounds. Cases of obscure abdominal pain, which may have been regarded for a while as neurotic, sometimes turn out to be due to abdominal aneurysm, which ruptures and causes death; weird pains in the chest may be called "pleurodynia," and in a few months the patient may be dead of a mediastinal growth; headaches may be regarded as trivial, or exaggerated by the patient and diagnosed as "cephalalgia"; yet now and then they will be due to a cerebral abscess. There is no need to raise the patient's fears, or those of the friends, whenever there are symptoms which *may* have a grave cause; in almost all cases it would be very unwise indeed to do so; but at the same time there seems to be a considerable tendency, especially amongst house-physicians and newly-qualified men, to pooh-pooh the patient's story of subjective symptoms, and to diagnose hysteria as often as possible instead of as seldom as possible. There is a great tendency to treat people suffering from neuroses as though they were malingering, whereas hysteria and neuroses are diseases, in that patients suffering from them are ill, and that it is the duty of the doctor to try and make them well. All organs have both a structure and a function—an anatomy and a physiology; they are liable to disorders of either or both. Neuroses are disorders of physiological function without disorder of anatomical structure; they are very difficult indeed to treat successfully, and they do not kill, and are therefore too often regarded as mere nuisances, which are best left alone. It is not, however, of this feature that we propose to give an example to-day, but rather to describe a case in which the diagnosis seemed to be neurosis, though the result proved it to be otherwise.

The patient was a well-developed, comely girl, aged 17, who came under observation for peculiar dyspnœa of a paroxysmal nature. She had been absolutely well until March 9, actively engaged in her work as a domestic servant, bright and happy as far as could be judged. She was not able to give any family history, for she was an orphan. She had lived healthily as far as was known. Suddenly on March 9 she had an attack of severe dyspnœa, without cyanosis, without stridor, without pain, without palpitations, without up and down movements of the larynx, but with a very rapid rate of respiration. She was quite sure that she had got no foreign body down the wrong way, and she herself could not account for the dyspnœa. She said she felt suffocated whilst the attack was on, and towards the end of it she had a little ineffectual cough. She was put to bed, and the paroxysm gradually passed off in about an hour. She got up again next day, and was at first able to work; but presently another precisely similar attack occurred, passing off when she went to bed; and the attacks repeated themselves as often as she got up, especially if people were watching her; when she was left quite alone she was able to move about like an ordinary person, free from dyspnœa, but whenever she knew she was watched a paroxysm came on with a noise that was not a true stridor, but was rather something between a wheeze and a groan at each respiratory effort. These attacks went on and on from March to August. The patient was examined carefully in all kinds of ways. The cardiac physical signs were perfectly normal. Nothing whatever could be found wrong with the lungs—during the paroxysms of dyspnœa there was a groaning sound heard all over the chest, and the expiratory part of the vesicular murmur was much prolonged, but equally so on both sides. The thyroid gland was apparently natural; there was nothing the matter with the urine; the larynx looked perfectly sound on laryngoscopic examination; there was no paresis of either cord, no structural deformity, no foreign body, no papilloma. The abdominal viscera were apparently natural. The knee jerks were exaggerated, but the rest of the nervous reflexes were normal.

The attacks were very similar to those sometimes seen in older patients with thoracic aneurysm or mediastinal new growth, so the chest was skiagraphed; absolutely no abnormality was seen with the *x*-rays. The blood examination for eosinophilia in support of a diagnosis of asthma was negative.

An enlargement of the thymus gland obstructing the bronchi was suggested as a possibility, and attempts to percuss out this gland were made without success; moreover, the dyspnœa was not influenced by the position of the patient; indeed, the attacks came on much more frequently when she was vertical than when she was lying down, whereas a large thymus gland obstructs the bronchi most in the lying posture, because the sternum is then at its closest approximation to the bodies of the vertebræ behind.

Syphilitic stenosis of a bronchus was suggested, and anti-syphilitic treatment was persisted with for many weeks without the slightest benefit; there was not a sign of any syphilitic lesion elsewhere, the patient had lived all her life in respectable surroundings, and was only 17, so that, especially in view of the failure of anti-syphilitic remedies, syphilis seemed put out of court.

As no organic lesion seemed to fit the case, and as the girl did not lose flesh, and seemed strong, robust, and pretty, it was thought that the trouble must be functional. Except for the attacks of sudden dyspnœa, which sometimes lasted a whole day, sometimes much less, she felt and looked perfectly well. Sometimes she seemed a little better, sometimes a little worse, but upon the whole there was no obvious change either way from March to August. The attacks were always brought on when she was up and about and watched; they sometimes came on when she was watched in bed, or examined with the stethoscope; but if left to herself in bed she would go for long times without an attack, though she always breathed rather more rapidly than normal persons do.

Towards the end of August she had one of her severe paroxysms of dyspnœa, with little or no cough; everyone expected it would pass off as the scores of others had done, but instead of that she suddenly became extremely cyanosed, and died asphyxiated.

At the autopsy there was a dense fibrous stenosis of the last inch of the trachea and the first inch of each of the bronchi, due almost certainly to a healed gummatous infiltration.

The reason why anti-syphilitic treatment had done no good was that the gummata had already been converted into irremovable fibrous tissue. The reason why there had been no very abnormal lung signs was that the lesion was symmetrical and bilateral, so that there was no means of obtaining a difference in vesicular murmur between the two sides of the chest; and the trouble had led neither to broncho-pneumonia nor to fibrosis of the lung with bronchiectasis.

There was no syphilitic lesion anywhere else in the body; indeed, except for the tracheal and bronchial stenosis, and for acute over-distension of the right side of the heart, all the viscera were normal.

